# 2433. Implementation of Midline Vascular Access Program and Reduction of Central Line-Associated Bloodstream Infections (CLABSI) in an Intensive Care Unit at a Community-Based Hospital

**DOI:** 10.1093/ofid/ofad500.2052

**Published:** 2023-11-27

**Authors:** E L T A I B A SAAD, Qingqing Meng, Mohammed Faris, Jonathan Stake

**Affiliations:** Ascension Saint Francis Hopsital, Evanston, Illinois; Ascension Saint Francis Hospital, Evanston, Illinois; Ascension Saint Francis Hopsital, Evanston, Illinois; Ascension Saint Francis Hopsital, Evanston, Illinois

## Abstract

**Background:**

Central venous catheter (CVC) placement is the most frequently performed procedure in acute care hospitals for various indications in multiple clinical settings. CVCs are the main source of hospital-acquired bloodstream infections in the United States (US). Central line-associated bloodstream infections (CLABSIs) constitute a significant contributor to gross healthcare expenditure in the US health system. Reduction of the duration of the CVCs is a key factor to decrease the risk of CLABSI.

**Methods:**

A retrospective study was conducted to compare the annual incidence of CLABSI rate, total CVC days, and device utilization ratio before and after the implementation of a midline vascular access program as an institutional-based intervention to reduce the CLABSI rate in the intensive care unit (ICU).

**Results:**

The midline vascular access program was started in June 2021. Two study groups were compared (Group 1 includes the study period from January 2019 to June 2021 and Group 2 from July 2021 to December 2022). CLABSI number decreased from 22 to nine. The CLABSI rate (measured as CLABSI number per 1000 CVC days) decreased by 52% (6.8% o 3.3%) (*P* < 0.05) . There was a reduction in the total CVC days (3198 days in Group 1 vs 2731 days in Group 2) but it was not significantly associated with the decline in the CLABSI rate (*P*=0.0702). A 60% significant reduction rate was observed in the device utilization ratio (measured as total CVS days/ total patients’ days) (0.6 to 0.3) (*P* < 0.001), which was significantly associated with the decrease in the CLABSI rate (*P*< 0.001).

**Table 1.**
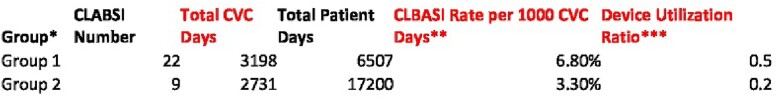
Results of study variables between the two study groups

**Figure 1.**
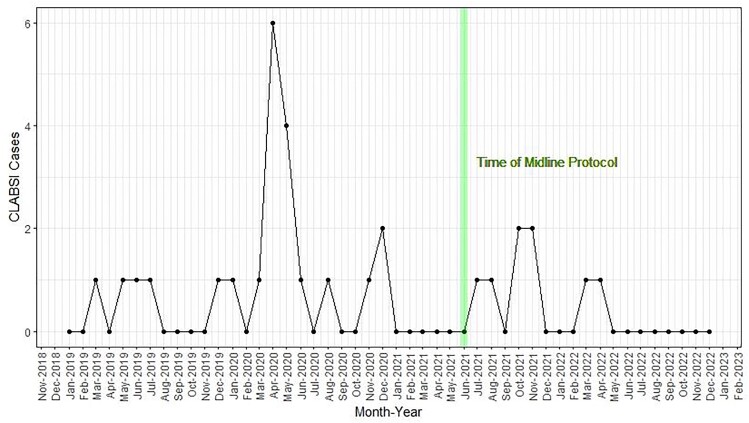
The monthly number of CLABSI infections during the study period.

**Conclusion:**

The midline vascular access program was associated with a reduction in the CLABSI rate per 1000 CVC days and the device utilization ratio in our ICU. The authors of this project are currently conducting a larger prospective case-control study to ascertain our preliminary study’s results which have some limitations attributed to its retrospective nature and sample size.

**Disclosures:**

**All Authors**: No reported disclosures

